# The Impact of COVID-19 on Cellular Factors Influencing Red Blood Cell Aggregation Examined in Dextran: Possible Causes and Consequences

**DOI:** 10.3390/ijms241914952

**Published:** 2023-10-06

**Authors:** Maciej Bosek, Tomasz Wybranowski, Marta Napiórkowska-Mastalerz, Jerzy Pyskir, Michał Cyrankiewicz, Małgorzata Pyskir, Marta Pilaczyńska-Cemel, Alicja Szołna-Chodór, Mateusz Wrembel, Stefan Kruszewski, Grzegorz Przybylski

**Affiliations:** 1 Department of Biophysics, Faculty of Pharmacy, Collegium Medicum in Bydgoszcz, Nicolaus Copernicus University in Toruń, 85-067 Bydgoszcz, Poland; mbosek@cm.umk.pl (M.B.);; 2 Department of Rehabilitation, Faculty of Health Sciences, Collegium Medicum in Bydgoszcz, Nicolaus Copernicus University in Toruń, 85-067 Bydgoszcz, Poland; gosia@cm.umk.pl; 3 Department of Lung Diseases, Neoplasms and Tuberculosis, Faculty of Medicine, Collegium Medicum in Bydgoszcz, Nicolaus Copernicus University in Toruń, 85-067 Bydgoszcz, Poland

**Keywords:** COVID-19, red blood cells aggregation, porosity of aggregates, pneumonia, blood rheology

## Abstract

Several studies have indicated that COVID-19 can lead to alterations in blood rheology, including an increase in red blood cell aggregation. The precise mechanisms behind this phenomenon are not yet fully comprehended. The latest findings suggest that erythrocyte aggregation significantly influences microcirculation, causes the formation of blood clots in blood vessels, and even damages the endothelial glycocalyx, leading to endothelial dysfunction. The focus of this research lies in investigating the cellular factors influencing these changes in aggregation and discussing potential causes and implications in the context of COVID-19 pathophysiology. For this purpose, the aggregation of erythrocytes in a group of 52 patients with COVID-19 pneumonia was examined in a 70 kDa Dextran solution, which eliminates the influence of plasma factors. Using image analysis, the velocities and sizes of the formed aggregates were investigated, determining their porosity. This study showed that the process of erythrocyte aggregation in COVID-19 patients, independent of plasma factors, leads to the formation of more compact, denser, three-dimensional aggregates. These aggregates may be less likely to disperse under circulatory shear stress, increasing the risk of thrombotic events. This study also suggests that cellular aggregation factors can be responsible for the thrombotic disorders observed long after infection, even when plasma factors have normalized. The results and subsequent broad discussion presented in this study can contribute to a better understanding of the potential complications associated with increased erythrocyte aggregation.

## 1. Introduction

Some studies have revealed increased aggregation of erythrocytes in patients with COVID-19 [1,2,3,4,5,6]. The precise mechanisms underlying this phenomenon have not yet been entirely elucidated. Understanding the factors that contribute to red blood cell (RBC) aggregation in the context of COVID-19 is critical because it can provide valuable information about the pathophysiology of the disease. The properties of red blood cells can significantly impact the aggregation process, changing their aggregability [7]. Although the debate on the influence of aggregation, especially the typical rouleau formation, in blood flow is still ongoing, it is accepted that excessive aggregation, particularly the formation of three-dimensional (3-D) structures, can disrupt normal blood flow and lead to health issues [8,9,10,11,12]. Investigations into the formation of linear, branched, and 3-D structures by red blood cells are still underway, as not all aspects of this complex process are fully understood yet [8,13,14,15,16,17,18]. The compactness of 3-D structures of erythrocytes refers to the degree of closeness or cohesion of erythrocyte rouleaux or individual erythrocytes in the aggregate structure. Greater compactness indicates a denser packing of erythrocytes in the aggregate, resulting in lower porosity. Porosity, a naturally occurring feature of 3-D aggregates, is particularly significant when considering the implications of the aggregation process of red blood cells in the body. The aggregation phenomenon is observed across various scientific and medical disciplines. The porosity of aggregates refers to the presence of spaces in their structure that are not filled by the elements that make them up. The porosity of the RBC aggregates determines their shear resistance and effective density, which, in turn, impacts the blood flow properties. Its estimation can, therefore, be crucial in the study of various pathological conditions [19].

Aggregation between erythrocytes depends on a number of factors, one of which is the surface composition of the cells. Glycoproteins and glycolipids, contained in erythrocytes, possess sialic acid residues. These residues confer negative charges to the surface of the cell, creating the so-called zeta potential. Zeta potential is a measure of the electrostatic repulsion between particles in a fluid. It represents the potential difference between the surface of the erythrocyte and the surrounding fluid. The electrostatic repulsion resulting from the zeta potential is a mechanism that prevents erythrocyte aggregation [20,21,22]; pH, temperature, and the presence of different salts in the environment can also impact the interactions between these cells, altering their adhesive force. In turn, the ability of erythrocytes to adhere to each other is influenced by their mechanical properties, including their shape, size, and flexibility. For example, healthy erythrocytes have a unique biconcave shape that assists in the formation of rouleau structures. In addition, erythrocyte aggregation is affected by the presence of adhesion mediators. Substances like fibrinogen proteins can promote adhesion between erythrocytes by forming bridges between the cells, according to the bridging model, or by creating an osmotic pressure that attracts cells to each other, according to the depletion model [23,24]. All these factors are interrelated and together affect the overall adhesive force between erythrocytes.

The majority of the research indicates the role of plasma as a factor influencing aggregation in COVID-19 [1,2,3,4,5]. Despite its importance, focusing solely on fibrinogen and other plasma factors can overlook the potential influence of cellular factors on erythrocyte aggregation [7], while in the context of COVID-19, it is necessary to fully understand the contribution of both plasma and cellular factors to changes in erythrocyte aggregation. In order to study the influence of cellular factors on aggregation, the influence of individual plasma factors should be eliminated. This can be achieved by investigating the aggregation of erythrocytes in Dextran at a constant concentration and molecular weight, simulating uniform plasma conditions. Dextran is often used to study the behavior of erythrocytes due to its ability to induce their aggregation. Depending on its molecular weight and concentration, it can simulate certain physiological or pathological conditions [25,26].

The aim of this study is to investigate the cellular factors affecting the aggregation of erythrocytes in COVID-19 pneumonia patients. We employed a novel technique that involves examining image sequences of settling objects to measure the velocity and sizes of RBC aggregates. Subsequently, using these parameters, we determined their structure and assessed their porosity. Using this approach, we aimed to gain a comprehensive understanding of the behavior of aggregates and its implications in various medical and rheological contexts.

## 2. Results

**Figure 1:**
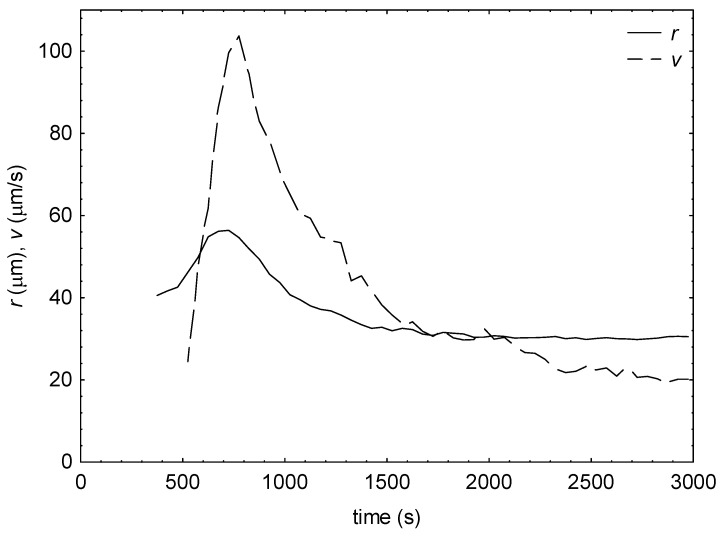
The aggregate radius (r) and velocity (v) as a function of time for COVID-19 patients upon admission to hospital.

An example of the time dependence of 3-D RBC aggregate radius and velocity was shown in Figure 1. As was expected, both values initially increased, peaked, and then decreased. The dynamics of this process for a given subject are well characterized by peak values of radius and velocity and times to these peaks.

**Figure 2:**
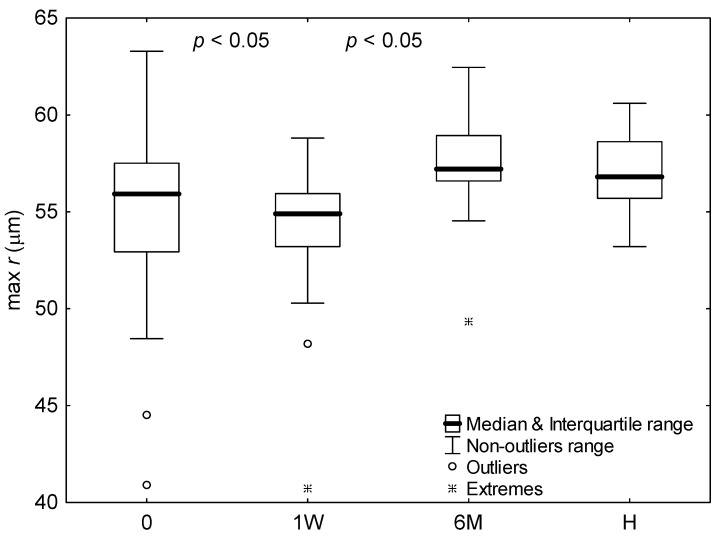
The peak aggregate radius for healthy subjects (H) and COVID-19 patients upon admission to hospital (0), after 1 week of hospitalization (1W), and 6 months after infection (6M). Values outside four and seven times the interquartile range were considered outliers and extremes, respectively. For significant differences between measurements or groups, the significance levels (*p*) for adjacent bars were shown.

**Figure 3:**
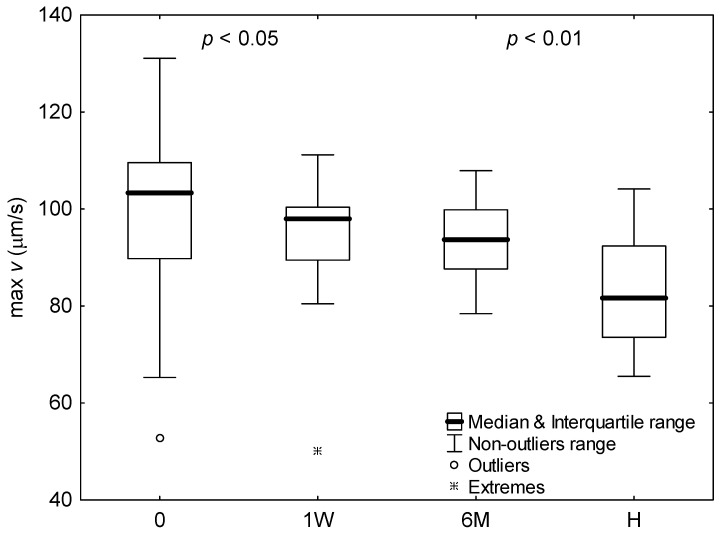
The peak aggregate velocity for healthy subjects (H) and COVID-19 patients upon admission to hospital (0), after 1 week of hospitalization (1W), and 6 months after infection (6M). Values outside four and seven times the interquartile range were considered outliers and extremes, respectively. For significant differences between measurements or groups, the significance levels (*p*) for adjacent bars were shown.

**Figure 4:**
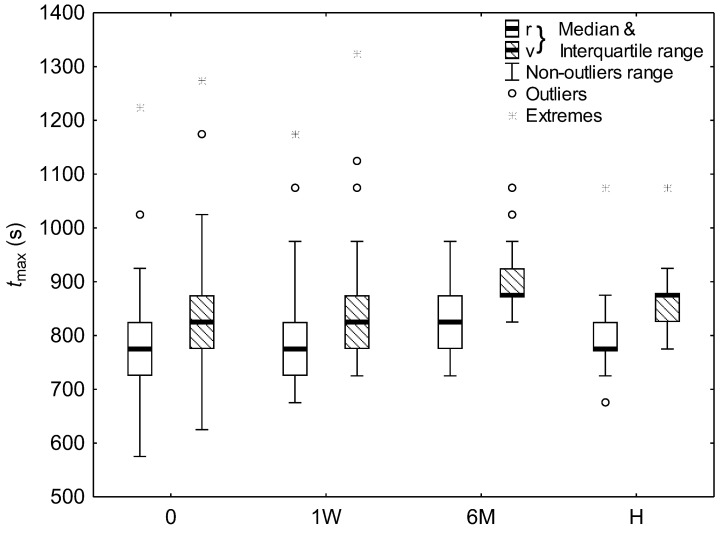
The time to peak aggregate radius and velocity for healthy subjects (H) and COVID-19 patients at admission to hospital (0), after 1 week hospitalization (1W), and 6 months after infection (6M). Values outside 4 and 7 times the interquartile range were considered outliers and extremes, respectively.

The changes in parameters of peak aggregate velocity and radius were summarized in Figure 2, Figure 3 and Figure 4 for subjects with COVID-19 upon admission to hospital, one week later, and six months after infection, as well as for healthy subjects.

It is seen that for subjects with COVID-19, the peak radius and velocity significantly decreased during hospitalization week (*p* < 0.05). The parameters, however, half a year after infection behaved differently. The aggregate radius significantly increased (*p* < 0.05), whereas velocity showed no significant difference. In comparison with healthy subjects, the aggregate radius for COVID-19 patients differed significantly only for measurement after hospitalization week (*p* < 0.01), where it was smaller. Whereas, the aggregate velocity for healthy subjects was significantly lower than that for COVID-19 patients for each measurement (*p* < 0.01). Time to peak aggregate velocity for COVID-19 patients was significantly greater half a year after infection than that upon admission to hospital (*p* < 0.05). Except that, time to peaks aggregate radius and velocity showed no significant differences.

**Figure 5:**
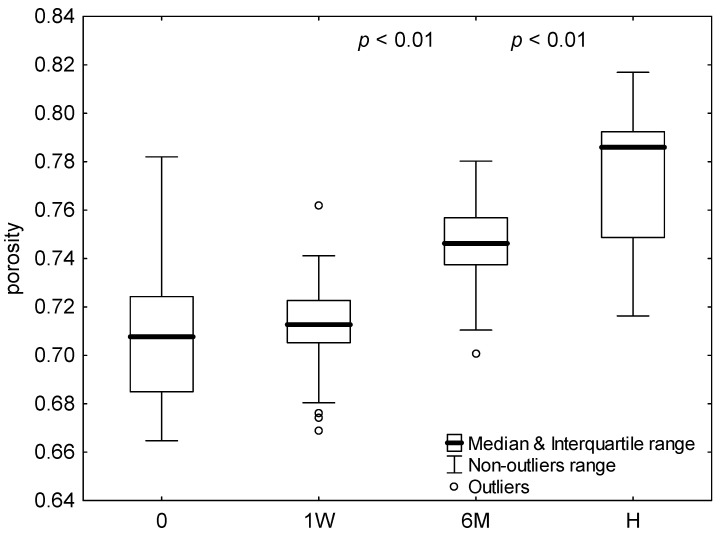
The aggregate porosity for healthy subjects (H) and COVID-19 patients at admission to hospital (0), after 1 week hospitalization (1W), and 6 months after infection (6M). Values outside four times the interquartile range were considered outliers. For significant differences between measurements or groups, the significance levels (*p*) for adjacent bars were shown.

Assuming no changes in erythrocyte density, the porosity of aggregates at their peak radius and velocity was calculated and shown in Figure 5. Aggregate porosity for hospitalized COVID-19 patients was significantly smaller than that for healthy subjects (*p* < 0.001) and did not change significantly during hospitalization week, whereas half a year after infection, the aggregate porosity significantly increased (*p* < 0.01); however, it was still significantly below the value for healthy subjects (*p* < 0.01).

## 3. Discussion

In this study, the parameters of erythrocyte aggregates were analyzed at the time when sedimentation overcame the aggregation, i.e., when the radius and velocity peaked. At this time, the largest aggregates began to leave the observed area due to sedimentation. This analysis provided information on the porosity of the formed aggregates as a combination of their radius and velocity. As mentioned, statistically significant changes in the porosity of erythrocyte aggregates in COVID-19 patients were observed only after half a year after infection. Following treatment and normalization of oxygen saturation via oxygen therapy, we did not notice changes in aggregate porosity. This may be due to the fact that changes in the cellular factors studied here require either membrane repair, which is not very efficient, or the replacement of erythrocytes in the bloodstream, which is a rather slow process since the lifespan of RBCs is about 120 days [27,28].

Half a year after infection, an increase in porosity was observed, indicating lower compactness. However, the observed values were still significantly lower than the typical values for the healthy group. This discrepancy may be attributed to the fact that patients admitted to the hospital had multiple comorbidities, as detailed in Table 1. Erythrocyte aggregation is often elevated by these diseases, as confirmed in several studies. Also, the infection itself may have worsened the patients’ conditions, often causing symptoms associated with the so-called “long COVID”. The obtained results suggest changes in erythrocyte aggregability in patients, resulting in an altered structure of the forming aggregates. Given that the time to peak velocity and radius in the observed field does not change significantly, the formation of denser and more complex aggregates occurs in the same time frame as under healthy conditions. This indicates that in the infectious environment of COVID-19, denser aggregates do not appear due to the prolonged process duration but due to the enhancement of the underlying mechanisms that promote erythrocyte aggregation. There are many studies reporting an increase in aggregation in COVID-19 patients. From our results, it can be concluded that the cellular factor of this increase does not simply result in larger aggregates but more compact ones. As it was well known, initially, the unique biconcave discoid morphology of the erythrocytes facilitates rouleaux formation, where cells aggregate in a stacked manner. However, for rouleaux composed of more than three erythrocytes, the shape begins to deviate increasingly from spherical, becoming increasingly energetically unfavorable [29]. In this situation, side-to-top connections appear, creating branched rouleaux. These structures can be formed directly or by reorganization of longer rouleaux [13]. Next, when aggregates become larger and more complex, they start to fill the available space more effectively, taking on an increasingly spherical shape. As a result, 3-D aggregates of densely packed erythrocytes are formed [30,31,32]. The transition from branched rouleaux to packed 3-D aggregates results in an increase in compactness of the aggregates, quantified as a reduction in their porosity. It is crucial to note that although a certain degree of RBC aggregation is natural and beneficial, abnormal states, such as the formation of 3-D structures with reduced porosity due to excessive aggregation, can lead to severe health complications, as observed in our COVID-19 patients during infection. In circulation, the factor that counteracts the formation of aggregates is the shear stress experienced by blood flowing in the vessels. The increased compactness of the aggregates indicates their greater stability, which may decrease the likelihood of disaggregation of these aggregates due to shear stress. In healthy subjects, the shear stress in the circulatory system is sufficient to separate the aggregate into individual erythrocytes [11,16,33]. Whereas, in COVID-19 patients, denser, more stable aggregates may not disaggregate under these conditions.

The reduction of the zeta potential of red blood cells, along with membrane damage and decreased elasticity, contributes to the increased tendency of erythrocytes to aggregate (aggregability). This may be caused by factors such as oxidative stress, direct interaction with the SARS-CoV-2 virus, hypoxia, or an immune response. It has been noted that oxidative stress plays a role in the pathophysiology of COVID-19 [4,34,35]. Reactive oxygen species (ROS) that are produced during oxidative stress can lead to oxidative damage of the lipids and proteins, which are components of erythrocytes [36,37]. We observed significantly higher levels of Advanced Oxidation Protein Products (AOPP) in patients during infection compared to the AOPP levels of the same patients at 6 months and 12 months post-infection, as well as compared to healthy individuals [38]. The lipid bilayer of the RBC membrane, which is rich in polyunsaturated fatty acids, is especially susceptible to peroxidation by ROS. This process involves the oxidation of lipids, leading to the production of malondialdehyde and other toxic byproducts [39]. Oxidative stress can affect the key 3-S proteins (Spectrin, Ankyrin, and Band 3 protein) present in erythrocytes, which are essential in shaping and maintaining the flexibility of red blood cells. Furthermore, research has demonstrated that oxidative stress-induced phosphorylation of Band 3 significantly influences erythrocyte shape, structure, and deformability [40,41,42]. Researchers emphasize the association of erythrocyte aggregation with Band 3, but the underlying mechanism has not been fully elucidated. Some studies showed a correlation between oxidative stress and sialic acid in the RBC membrane [43,44]. Evidence is emerging to suggest that COVID-19 can cause structural protein damage and membrane lipid remodeling in red blood cells [45]. Oxidative effects on glycophorins may lead to the loss or cross-link of sialic acid residues, which may reduce the zeta potential of erythrocytes, increasing their aggregation [44]. Although no studies have yet been conducted regarding the level of sialic acid in COVID-19 cases, certain conclusions can be drawn based on other pathological conditions where increased oxidative stress, as well as increased erythrocyte aggregation, have been observed. For example, in sepsis, a decrease in the sialic acid content within the RBC membrane has been found [44]. Furthermore, there is convincing evidence indicating that the quantity of sialic acid in erythrocytes decreases with human age and in patients with diabetes and hypercholesterolemia [43,46,47,48,49].

Other changes in the RBCs population that may affect their aggregation have also been observed in patients with COVID-19. Under conditions of hypoxia, such as those found in severe pneumonia caused by bacterial infection or related to COVID-19, the kidneys react by increasing the production of erythropoietin. This hormone stimulates the bone marrow to generate more red blood cells to enhance oxygen transport to the body’s tissues. As a result, there is an increase in the number of reticulocytes, which are newly formed RBCs that differ from mature ones [50]. They are usually more spherical and less flexible, and their cell membrane contains remnants of the endoplasmic reticulum [51].

Another example is the specific effect of interleukins on RBCs. In acute inflammation, cytokines such as interleukin-1β, interleukin-6, and interleukin-8 (IL-8) are released by immune cells at the site of inflammation. Cytokine storm, also known as cytokine release syndrome, is an excessive and dysregulated immune response to infection that can occur in some patients with COVID-19 [52]. A study by Bester et al. suggests that an increased level of IL-8 leads to RBC membrane deformation [53].

SARS-CoV-2 can interact with erythrocytes in various ways. First, the virus can directly infect cells of the hematopoietic system, including bone marrow stem cells. Due to the presence of angiotensin-converting enzyme-2 (ACE2) receptors on erythroid precursors, it is postulated that SARS-CoV-2 may potentially impact erythropoiesis. One of the potential effects of this interaction is to change the surface or structural properties of newly formed erythrocytes. This may result in abnormal or increased aggregation of erythrocytes. There is also emerging evidence suggesting that the virus may directly interact with RBCs, possibly through the spike protein’s binding to Band 3 protein or CD147 [54,55]. This hypothesis is based on computational or in vitro studies and awaits confirmation in vivo in the context of actual COVID-19 patients. The suggested interaction could potentially influence the biophysical properties of RBCs, such as zeta potential, leading to alterations in their tendency to aggregate and promote hemagglutination [56]. However, considering the typically low viral load in blood observed in COVID-19 patients, a direct influence of SARS-CoV-2 on RBC aggregation through this mechanism seems unlikely.

Our results indicate a significant disruption in the formation of aggregates towards more compact and stable 3-D structures in patients with COVID-19-related pneumonia. It is plausible to hypothesize that erythrocyte aggregates may manifest in vessels in COVID-19 patients where they are typically absent. This raises important questions about the potential implications for disease pathogenesis. Understanding the mechanisms behind these disturbances is crucial to elucidating the impact on respiratory symptoms, disease severity, and the development of complications, such as acute respiratory distress syndrome (ARDS) or multiorgan failure. When considering the symptoms associated with COVID-19, the following clinical implications of these more compact erythrocyte aggregates are possible: altered rheological properties, impaired microcirculation, increased risk of thrombosis, and endothelial dysfunction.

Excessive aggregation of red blood cells can impair microcirculation, decrease oxygen transport to tissues, or even cause substantial obstruction of tiny vessels, such as capillaries, leading to a number of complications [10,57,58,59,60,61]. This may include ischemia of internal organs or limbs. This could potentially contribute to the development of organ dysfunction and complications observed in severe cases of COVID-19, such as ARDS or multiorgan failure. Ischemia in limb tissues can lead to various symptoms, including pain. This pain may be the result of hypoxia and irritation of pain receptors in the tissues, which can manifest as a sensation of burning, tingling, heaviness, or stiffness in the limbs. These symptoms are frequently observed in patients with COVID-19 [62,63].

Aggregation of RBCs can influence the rheological properties of blood, such as viscosity and flow dynamics. Alterations in blood rheology can affect overall circulation and may contribute to the systemic manifestations observed in severe COVID-19 cases. Increased blood viscosity, potentially due to an increase in erythrocyte aggregation, has been suggested to contribute to ischemic heart disease and stroke [64]. The impact of RBC aggregation on resistance, velocity, and viscosity in various vessels is widely discussed [8,9,13,14,15,16,65,66]. Aggregation of RBCs tends to increase the viscosity of blood under low-shear conditions [12,67]. When considering blood as it flows through the vessels in the body, it is important to note that it does not behave like a simple, homogeneous fluid. Instead, it exhibits a variety of complex behaviors due to the presence of different components, including RBCs, white blood cells, platelets, and plasma. In larger vessels, the velocity of blood is greater in the center of the vessel than near the walls. This is due to the fact that frictional forces between the blood and the vessel walls slow down the flow close to the walls, creating a velocity gradient. This is known as the “no-slip condition” in fluid dynamics. Studies based on a mathematical model suggest that erythrocyte aggregation generates a different flow velocity profile for blood than that for Poiseuille flow. The velocity, instead of the maximum in the center of the vessel, is approximately constant at some distance from the walls of the vessel [68]. However, when blood flows through smaller vessels, RBCs naturally move towards the center of the blood vessels. This movement leads to the creation of a cell-free, plasma-rich zone along the vessel walls. This segregation results in a lower viscosity of the blood in the marginal region than in the bulk flow. This is because the viscosity of plasma is much lower than the viscosity of a concentrated suspension of red blood cells. Especially in small vessels, axial accumulation of red blood cells is further enhanced by their aggregation [8,12,67,69,70,71]. When blood flows from a larger vessel into smaller branching vessels, plasma “skims off” into the smaller branches, leaving a higher concentration of red blood cells in the parent vessel. Increasing aggregation enhances this process, additionally lowering the hematocrit in the microvessels [8]. Interestingly, in in vitro experiments with the blood from COVID-19 patients, aggregation occurred in the microfluidic channel. This contrasted with virtually no aggregation of erythrocytes from the blood of healthy individuals [72]. It has also been observed that with increased aggregation, more force is required to separate the aggregates into individual red blood cells as blood flows into the capillaries from larger vessels. The cause of this phenomenon may be less porous, hence more compact and packed 3-D aggregates observed in this study in COVID-19 patients.

Erythrocyte aggregation can promote thrombosis in several ways. First, increasing blood viscosity can impede blood circulation and lead to stasis or slowing of blood flow. In addition to endothelial injury and hypercoagulability, decreased blood flow is one of the factors known as Virchow’s Triad, considered to be the key factor contributing to the formation of a blood clot [73]. Postcapillary venules are the blood vessels most susceptible to RBC aggregation. Due to their larger size, postcapillary venules provide ample space for RBCs to come together and form aggregates. Additionally, the low shear stress in these vessels reduces the force exerted on the RBCs, allowing them to stick together more easily [74]. It is well known that patients with COVID-19 are at heightened risk of developing blood clots, including deep vein thrombosis (DVT), usually in the legs [75,76]. One of the primary concerns with DVT is the risk of the clot breaking loose and traveling to the lungs, which can cause pulmonary embolism, a life-threatening condition [77]. Pulmonary embolism can block blood flow to the lungs, leading to low blood oxygen levels, lung impairment, and consequently, even damage to other organs in the body. It is worth noting here that in one study, artificially inducing RBC aggregation has been found to promote thrombosis in the femoral veins of rabbits [78]. Second, other studies have hypothesized that enhanced red blood cell aggregation may not only stabilize clots but may also trigger platelet activation, further promoting clot formation [75,79,80]. Moreover, the already mentioned axial accumulation of erythrocytes during blood flow, in addition to plasma, pushes platelets and white blood cells toward the vessel walls. Increased aggregation of erythrocytes, e.g., during the initial stages of the inflammatory response or when a blood vessel is damaged, can additionally promote this phenomenon [11,81,82,83,84]. A positive correlation was found between the degree of adhesion of 1 µm microparticles, imitating leukocytes and platelets, to the vessel wall and the extent of RBC aggregation induced by Dextran [85]. Erythrocyte aggregation, by changing the shear stress acting on endothelial cells, may also indirectly influence platelet adhesion to vessel walls by modulating nitric oxide production. It is well established that nitric oxide, which exhibits anti-thrombotic effects, plays a key role in inhibiting blood clot formation. During the flow of blood through small vessels, the aggregation of erythrocytes can lead to a decrease in blood viscosity in the marginal zone—this reduction in viscosity results in lower shear stress experienced by the endothelial cells lining the vessel walls. As a consequence of this altered shear stress, there is a decrease in nitric oxide production by endothelial cells [71,86,87,88,89,90,91,92].

Emerging evidence shows that RBC aggregation can cause damage to the endothelial cell glycocalyx [72]. This finding is quite unexpected and has not been previously reported. The researchers suggest that in patients with COVID-19, erythrocyte aggregates can be observed within narrow vessels even during blood flow, and these aggregates mechanically damage the endothelium, further exacerbating the vascular complications associated with the disease. To support these findings, a series of experiments were carried out using microfluidic devices coated with human umbilical vein endothelial cells and patient blood samples. Regardless of the validity of these assertions, other studies have indeed shown damage or alterations to the glycocalyx, such as shedding or enzymatic cleavage in COVID-19 patients [93,94,95]. Damage to the endothelial glycocalyx can significantly impair the functionality of the vascular endothelium and directly contribute to the increased risk of thrombosis [96,97]. Furthermore, endothelial dysfunction is associated with various cardiovascular complications and may contribute to the development of multiorgan failure in severe cases of COVID-19.

In addition to the cellular factors investigated in this study, the aggregation of erythrocytes in vivo is also influenced by plasma factors, specifically an increase in fibrinogen concentration and a rise in high-molecular-weight AOPPs. In our previous work, we demonstrated that the porosity of the formed aggregates decreases with increasing concentration of Dextran, regardless of its various molecular weights [98]. This suggests that in plasma, the effects described above may be magnified.

It is worth noting that there have been many reports of an increase in the incidence of venous thromboembolism (VTE), even several months after COVID-19 infection [99,100]. The reason for this may be the increased erythrocyte ability to aggregate, which, in contrast to the plasma factors of aggregation, i.e., acute phase proteins and fibrinogen, may persist long after the infection, lasting throughout the lifespan of altered erythrocytes, i.e., up to 120 days.

## 4. Materials and Methods

### 4.1. The General Characteristics of the Studied Patients

The patients included in this study were hospitalized due to COVID-19-pneumonia in the Department of Lung Diseases, Neoplasms, and Tuberculosis of the Regional Center of Pulmonology in Bydgoszcz, Poland, and enrolled from April to December 2021. COVID-19 infection was confirmed by a positive reverse transcription-polymerase chain reaction (RT-PCR) (Becton, Dickinson, and Company, Franklin Lakes, NJ, USA) test result from a nasopharyngeal swab according to World Health Organization criteria [101]. In this reaction, reagents target RNA from the nucleocapsid phosphoprotein gene (N1 and N2 regions) of the SARS-CoV-2 coronavirus. Radiographic imaging (HRCTs with a 64-slice Siemens Somatom Sensation (Siemens Healthcare, Erlangen, Germany) system with a slice thickness ≤ 0.5 mm) or chest X-ray were eligible for enrolment. All of the patients had a blood oxygen saturation below 94% while breathing ambient air. Exclusion criteria were receiving continuous positive airway pressure, bilevel positive airway pressure, or mechanical ventilation. The baseline characteristics of the studied patients are shown in Table 1. The group consisted of 11 women and 43 men, and the median age of all of the patients was 65 years. Most patients were previously diagnosed with one or more comorbidities. In our participants, the prevalent comorbidities were cardiovascular diseases (20.4%), diabetes (25.9%), previous lung diseases (11.1%), cancer (7.4%), chronic kidney disease (7.4%), thyroid diseases (7.4%), and neurological diseases (25.9%). Basic laboratory tests were performed for all cases included. The mean lung involvement score assessed by the application of CT pneumonia analysis was 28%. In this study, we also included the control group of 15 subjects who were considered healthy.

### 4.2. Blood Collection and Preparation of the Sample

The blood collection occurred three times: upon admission to the hospital, one week later, and six months after infection. The characteristics of the study groups are shown in Table 1. The blood from each participant was collected into EDTA-containing tubes and was immediately mixed and centrifuged at 3000 rpm at 4 °C for 5 min to separate RBCs from plasma and buffy coat. Then, RBCs were washed three times by centrifuging in a solution of phosphate-buffered saline (PBS). Before each measurement, suspension of RBCs at hematocrit 0.05 in a solution of Dextran 70 kDa in PBS at a concentration of 3 g/dL was prepared. Next, the sample was immediately shaken and injected into a rectangular glass-walled container of 18 mm width × 23 mm height × 1 mm depth to conduct a measurement.

### 4.3. Measurement

Images of the sample uniformly illuminated by a white LED were recorded every second using a CCD camera (The Imaging Source, Bremen, Germany) for 50 min. The recorded section was in the center of the sample and had a size of 4 mm × 3 mm (1280 × 960 pixels). The measurement was carried out at a temperature of about 22 °C.

### 4.4. Data Analysis

**Figure 6:**
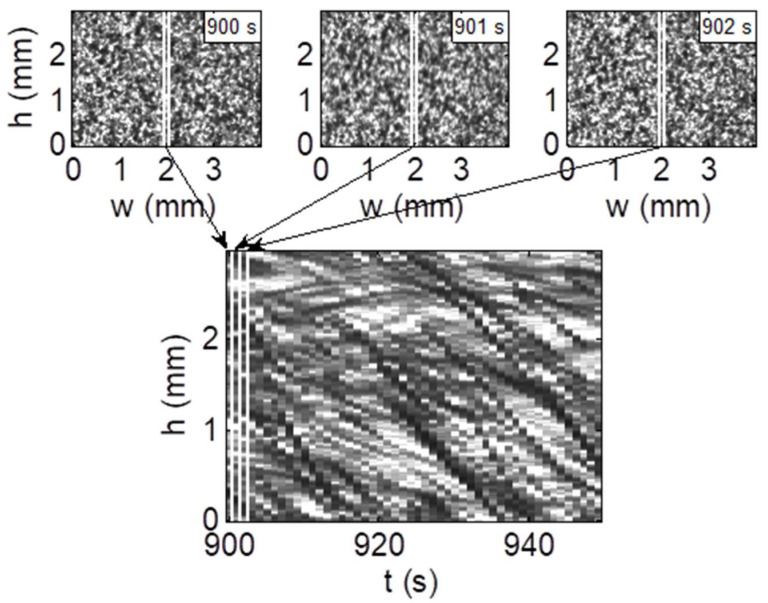
The consecutive images of red blood cell suspension (**top**) and the image time sequence composed from them (**bottom**) for COVID-19 patients upon admission to hospital.

The image analysis method used in this study involves analyzing a time series of images to estimate the velocity and size of three-dimensional RBC aggregates. These procedures are described in more detail in the publication [98,102]. To analyze the aggregate movement, the time sequences of one-pixel-wide clippings for a given column of the sample from 50 consecutive images were composed (Figure 6). The vertical movement of an aggregate within a given column is seen in this time sequence as a line whose slope gives the velocity of this aggregate. To obtain the line representing the mean movement of all aggregates, two-dimensional autocorrelations of time sequences for each column were calculated and averaged. This line was obtained along the peak of this function using the least squares method in inverse space, and its slope gave the mean aggregate velocity. This autocorrelation as a function of sample height shift for a time shift equal to zero also allows for an estimation of the aggregate size. The shift at which an exponential fit to this function decreased *e*-times was assumed as the mean aggregate radius.

The velocity and radius of the aggregates allow for estimating the aggregate porosity. In this study, porosity refers to the proportion of volume filled by the solution between erythrocytes within an aggregate compared to the total volume of the aggregate itself. From the balance of forces acting on aggregates and taking into account the hindrance effect obtained by Richardson and Zaki [103,104], the porosity is given by:

$$p=1-\frac{9\mu }{2g\left(\rho_{e}-\rho_{s}\right){\left(1-H\right)}^{4.65}}\frac{v_{a}}{r_{a}^{2}}$$

where *μ* = 1.277 mPas is the viscosity of the solution, *ρ_e_* = 1110 μg/mm^3^ and *ρ_s_* = 1027 μg/mm^3^ are the density of the erythrocyte and solution, respectively, *g* = 9.81 m/s^2^ is the gravity acceleration, *H* = 0.05 is the hematocrit of the suspension, and *v_a_* and *r_a_* are the average velocity and radius of the aggregates.

### 4.5. Statistical Analysis

In order to select a test to examine differences between samples, the normality of the distributions of the measured parameters was examined using the Shapiro–Wilk test. Since some of them did not have a normal distribution, the statistical significance of these differences was determined using the Mann–Whitney U test between independent samples and the Wilcoxon test between dependent samples. The results of the tests were accepted as statistically significant when *p* < 0.05.

## 5. Conclusions

The comprehensive analysis of the causes and consequences of increased erythrocyte aggregation and reduced aggregate porosity in individuals with COVID-19 conducted in this study can contribute to a better understanding of the pathophysiology of this disease. Our study underscores the necessity of considering cellular aggregation factors, emphasizing their crucial role in investigating erythrocyte aggregation. We observed the effect of these factors on increasing the compactness of the aggregates. It is essential to emphasize that the compactness of aggregates significantly impacts their stability and behavior in blood. It is particularly relevant in the context of microcirculation disorders, deep vein thrombosis, endothelial dysfunction, and oxygen transport in COVID-19. These findings provide the foundation for further research and potential advancements in the field of erythrocyte-related disorders. Our study also implicates cellular factors of aggregation as the cause of thrombotic disorders that persist long after infection, even when plasma factors of aggregation have already normalized.

## Figures and Tables

**Table 1:** Baseline characteristics of the studied groups.

	Study Group—COVID-19			Control Group
	Upon Admission	After One Week	After 6 Months	
Number	54	33	23	15
Median and range of age (years)	65 (37–98)	68 (37–98)	62 (46–87)	42 (25–61)
Gender				
Women	11 (20.4%)	8 (24.2%)	4 (17.4%)	12 (80%)
Men	43 (79.6%)	25 (75.8%)	19 (82.6%)	3 (20%)
Smoking				
Yes	12 (22.2%)	4 (12.1%)	4 (17.4%)	3 (20%)
No	42 (77.8%)	29 (87.9%)	19 (82.6%)	12 (80%)
Common symptoms:				-
Dyspnea	44 (81.5%)	26 (78.8%)	17 (73.9%)	
Cough	42 (77.8%)	26 (78.8%)	17 (73.9%)	
Fever	48 (88.9%)	29 (87.9%)	22 (95.7%)	
Myalgia and arthralgia	16 (29.6%)	10 (30.3%)	9 (39.1%)	
Changes in the sense of smell and/or taste	8 (14.8%)	3 (9.1%)	3 (13%)	
Common comorbidities:				-
Cardiovascular diseases	11 (20.4%)	4 (12.1%)	3 (13%)	
Type 2 diabetes	14 (25.9%)	8 (24.2%)	3 (13%)	
Previous lung diseases	6 (11.1%)	3 (9.1%)	2 (8.7%)	
Cancer	4 (7.4%)	2 (6.1%)	2 (8.7%)	
Chronic kidney disease	4 (7.4%)	2 (6.1%)	0 (0%)	
Thyroid diseases	4 (7.4%)	3 (9.1%)	2 (8.7%)	
Neurological diseases	14 (25.9%)	4 (12.1%)	7 (30.4%)	
Treatment:		-	-	-
Convalescent plasma	13 (24.1%)			
Remdesivir	22 (40.7%)			
Tocilizumab	16 (29.6%)			
Steroids	54 (100%)			
Antibiotics	53 (98.1%)			
Heparin	52 (96.3%)			
Amantadine	7 (13%)			
Oxygen supplementation	54 (100%)			
Median and IQR of acute phase proteins:			-	-
CRP (mg/L)	77.5 (40–130)	-		
Procalcitonin (ng/mL)	0.07 (0.05–0.14)	-		
Albumin (g/L)	34 (31–36)	34 (31–36)		

## Data Availability

The data presented in this study are available on request from the corresponding author.

## References

[1] Renoux C., Fort R., Nader E., Boisson C., Joly P., Stauffer E., Robert M., Girard S., Cibiel A., Gauthier A. (2021). Impact of COVID-19 on Red Blood Cell Rheology. Br. J. Haematol..

[2] Bizjak D.A., John L., Matits L., Uhl A., Schulz S.V.W., Schellenberg J., Peifer J., Bloch W., Weiß M., Grüner B. (2022). SARS-CoV-2 Altered Hemorheological and Hematological Parameters during One-Month Observation Period in Critically Ill COVID-19 Patients. Int. J. Mol. Sci..

[3] Ifrig E., Fibben K., Silvestri G., Maier C., Lam W. (2021). Fibrinogen-RBC Interactions Play a Key Role in COVID-19-Associated Endothelial Dysfunction. Chest.

[4] Russo A., Tellone E., Barreca D., Ficarra S., Laganà G. (2022). Implication of COVID-19 on Erythrocytes Functionality: Red Blood Cell Biochemical Implications and Morpho-Functional Aspects. Int. J. Mol. Sci..

[5] Nader E., Nougier C., Boisson C., Poutrel S., Catella J., Martin F., Charvet J., Girard S., Havard-Guibert S., Martin M. (2022). Increased Blood Viscosity and Red Blood Cell Aggregation in Patients with COVID-19. Am. J. Hematol..

[6] Grau M., Ibershoff L., Zacher J., Bros J., Tomschi F., Diebold K.F., Predel H.G., Bloch W. (2022). Even Patients with Mild COVID-19 Symptoms after SARS-CoV-2 Infection Show Prolonged Altered Red Blood Cell Morphology and Rheological Parameters. J. Cell. Mol. Med..

[7] Rampling M.W., Meiselman H.J., Neu B., Baskurt O.K. (2004). Influence of Cell-Specific Factors on Red Blood Cell Aggregation. Biorheology.

[8] Baskurt O.K., Meiselman H.J. (2013). Erythrocyte Aggregation: Basic Aspects and Clinical Importance. Clin. Hemorheol. Microcirc..

[9] Baskurt O.K., Meiselman H.J. (2008). RBC Aggregation: More Important than RBC Adhesion to Endothelial Cells as a Determinant of in Vivo Blood Flow in Health and Disease. Microcirculation.

[10] Baskurt O.K., Meiselman H.J. (2003). Blood Rheology and Hemodynamics. Seminars in Thrombosis and Hemostasis.

[11] Baskurt O.K., Meiselman H.J. (2007). Hemodynamic Effects of Red Blood Cell Aggregation. Indian J. Exp. Biol..

[12] FÅHRAEUS R. (1958). The Influence of the Rouleau Formation of the Erythrocytes on the Rheology of the Blood. Acta Med. Scand..

[13] Wagner C., Steffen P., Svetina S. (2013). Aggregation of Red Blood Cells: From Rouleaux to Clot Formation. C. R. Phys..

[14] Svetina S., Ziherl P. (2008). Morphology of Small Aggregates of Red Blood Cells. Bioelectrochemistry.

[15] Kendall K., Stainton C. (2001). Adhesion and Aggregation of Fine Particles. Powder Technol..

[16] Skalak R. (1984). Aggregation and Disaggregation of Red Blood Cells. Biorheology.

[17] Flormann D., Aouane O., Kaestner L., Ruloff C., Misbah C., Podgorski T., Wagner C. (2017). The Buckling Instability of Aggregating Red Blood Cells. Sci. Rep..

[18] Dasanna A.K., Darras A., John T., Gompper G., Kaestner L., Wagner C., Fedosov D.A. (2022). Erythrocyte Sedimentation: Effect of Aggregation Energy on Gel Structure during Collapse. Phys. Rev. E.

[19] Alexy T., Detterich J., Connes P., Toth K., Nader E., Kenyeres P., Arriola-Montenegro J., Ulker P., Simmonds M.J. (2022). Physical Properties of Blood and Their Relationship to Clinical Conditions. Front. Physiol..

[20] Eylar E.H., Madoff M.A., Brody O.V., Oncley J.L. (1962). The Contribution of Sialic Acid to the Surface Charge of the Erythrocyte. J. Biol. Chem..

[21] Fernandes H.P., Cesar C.L., Barjas-Castro M.d.L. (2011). Electrical Properties of the Red Blood Cell Membrane and Immunohematological Investigation. Rev. Bras. Hematol. Hemoter..

[22] Hughes M.P., Kruchek E.J., Beale A.D., Kitcatt S.J., Qureshi S., Trott Z.P., Charbonnel O., Agbaje P.A., Henslee E.A., Dorey R.A. (2021). Vm-Related Extracellular Potentials Observed in Red Blood Cells. Sci. Rep..

[23] Lopes C.S., Curty J., Carvalho F.A., Hernández-Machado A., Kinoshita K., Santos N.C., Travasso R.D.M. (2023). A Mathematical Model of Fibrinogen-Mediated Erythrocyte–Erythrocyte Adhesion. Commun. Biol..

[24] Semenov A.N., Lugovtsov A.E., Shirshin E.A., Yakimov B.P., Ermolinskiy P.B., Bikmulina P.Y., Kudryavtsev D.S., Timashev P.S., Muravyov A.V., Wagner C. (2020). Assessment of Fibrinogen Macromolecules Interaction with Red Blood Cells Membrane by Means of Laser Aggregometry, Flow Cytometry, and Optical Tweezers Combined with Microfluidics. Biomolecules.

[25] Pribush A., Zilberman-Kravits D., Meyerstein N. (2007). The Mechanism of the Dextran-Induced Red Blood Cell Aggregation. Eur. Biophys. J..

[26] Vijayaraghavan M., Chatterjee S., Sumantran V.N., Jayavelu T. (2022). Revisiting Dextran Effect on Red Blood Cell to Understand the Importance of Rouleaux Distribution and Red Blood Cell-Endothelial Cell Adhesion. Biomass Convers. Biorefin..

[27] Barshtein G. (2022). Biochemical and Biophysical Properties of Red Blood Cells in Disease. Biomolecules.

[28] Thiagarajan P., Parker C.J., Prchal J.T. (2021). How Do Red Blood Cells Die?. Front. Physiol..

[29] Szolna-Chodór A., Bosek M., Grzegorzewski B. (2015). Kinetics of Red Blood Cell Rouleaux Formation Studied by Light Scattering. J. Biomed. Opt..

[30] Fabry T.L. (1987). Mechanism of Erythrocyte Aggregation and Sedimentation. Blood.

[31] Kaliviotis E. (2015). Mechanics of the Red Blood Cell Network. J. Cell. Biotechnol..

[32] Pribush A., Meyerstein D., Meyerstein N. (2010). The Mechanism of Erythrocyte Sedimentation. Part 1: Channeling in Sedimenting Blood. Colloids Surf. B Biointerfaces.

[33] Semenov A., Lugovtsov A., Ermolinskiy P., Lee K., Priezzhev A. (2022). Problems of Red Blood Cell Aggregation and Deformation Assessed by Laser Tweezers, Diffuse Light Scattering and Laser Diffractometry. Photonics.

[34] Chernyak B.V., Popova E.N., Prikhodko A.S., Grebenchikov O.A., Zinovkina L.A., Zinovkin R.A. (2020). COVID-19 and Oxidative Stress. Biochemistry.

[35] Sadeq H., Daabo H. (2022). Role of Oxidative Stress in Pathogenesis and Severity of COVID-19 Infection: Case-Control Study in Iraq. J. Life Bio Sci. Res..

[36] Janz D.R., Ware L.B. (2015). The Role of Red Blood Cells and Cell-Free Hemoglobin in the Pathogenesis of ARDS. J. Intensive Care.

[37] Gyawali P., Richards R.S., Bwititi P.T., Nwose E.U. (2015). Association of Abnormal Erythrocyte Morphology with Oxidative Stress and Inflammation in Metabolic Syndrome. Blood. Cells. Mol. Dis..

[38] Wybranowski T., Napiórkowska M., Bosek M., Pyskir J., Ziomkowska B., Cyrankiewicz M., Pyskir M., Pilaczyńska-Cemel M., Rogańska M., Kruszewski S. (2022). Study of Albumin Oxidation in COVID-19 Pneumonia Patients: Possible Mechanisms and Consequences. Int. J. Mol. Sci..

[39] Spengler M.I., Svetaz M.J., Leroux M.B., Bertoluzzo S.M., Parente F.M., Bosch P. (2014). Lipid Peroxidation Affects Red Blood Cells Membrane Properties in Patients with Systemic Lupus Erythematosus. Clin. Hemorheol. Microcirc..

[40] Becatti M., Marcucci R., Gori A.M., Mannini L., Grifoni E., Alessandrello Liotta A., Sodi A., Tartaro R., Taddei N., Rizzo S. (2016). Erythrocyte Oxidative Stress Is Associated with Cell Deformability in Patients with Retinal Vein Occlusion. J. Thromb. Haemost..

[41] Remigante A., Morabito R., Marino A. (2021). Band 3 Protein Function and Oxidative Stress in Erythrocytes. J. Cell. Physiol..

[42] Carelli-Alinovi C., Dinarelli S., Sampaolese B., Misiti F., Girasole M. (2019). Morphological changes induced in erythrocyte by amyloid beta peptide and glucose depletion: A combined atomic force microscopy and biochemical study. Biochim. Biophys. Acta Biomembr..

[43] Shahvali S., Shahesmaeili A., Sanjari M., Karami-Mohajeri S. (2020). The Correlation between Blood Oxidative Stress and Sialic Acid Content in Diabetic Patients with Nephropathy, Hypertension, and Hyperlipidemia. Diabetol. Int..

[44] Piagnerelli M., Boudjeltia K.Z., Brohee D., Piro P., Carlier E., Vincent J.L., Lejeune P., Vanhaeverbeek M. (2003). Alterations of Red Blood Cell Shape and Sialic Acid Membrane Content in Septic Patients. Crit. Care Med..

[45] Thomas T., Stefanoni D., Dzieciatkowska M., Issaian A., Nemkov T., Hill R.C., Francis R.O., Hudson K.E., Buehler P.W., Zimring J.C. (2020). Evidence of Structural Protein Damage and Membrane Lipid Remodeling in Red Blood Cells from COVID-19 Patients. J. Proteome Res..

[46] Mehdi M.M., Singh P., Rizvi S.I. (2012). Erythrocyte Sialic Acid Content during Aging in Humans: Correlation with Markers of Oxidative Stress. Dis. Markers.

[47] Kumar D., Rizvi S.I. (2013). Erythrocyte Membrane Bound and Plasma Sialic Acid during Aging. Biologia.

[48] Rogers M.E., Williams D.T., Niththyananthan R., Rampling M.W., Heslop K.E., Johnston D.G. (1992). Decrease in Erythrocyte Glycophorin Sialic Acid Content Is Associated with Increased Erythrocyte Aggregation in Human Diabetes. Clin. Sci..

[49] Hadengue A., Razavian S.M., Del-Pino M., Simon A., Levenson J. (1996). Influence of Sialic Acid on Erythrocyte Aggregation in Hypercholesterolemia. Thromb. Haemost..

[50] Li H., Yang J., Chu T.T., Naidu R., Lu L., Chandramohanadas R., Dao M., Karniadakis G.E. (2018). Cytoskeleton Remodeling Induces Membrane Stiffness and Stability Changes of Maturing Reticulocytes. Biophys. J..

[51] Stevens-Hernandez C.J., Flatt J.F., Kupzig S., Bruce L.J. (2022). Reticulocyte Maturation and Variant Red Blood Cells. Front. Physiol..

[52] Soma P., Bester J. (2022). Pathophysiological Changes in Erythrocytes Contributing to Complications of Inflammation and Coagulation in COVID-19. Front. Physiol..

[53] Bester J., Pretorius E. (2016). Effects of IL-1β, IL-6 and IL-8 on Erythrocytes, Platelets and Clot Viscoelasticity. Sci. Rep..

[54] Cosic I., Cosic D., Loncarevic I. (2020). RRM Prediction of Erythrocyte Band3 Protein as Alternative Receptor for SARS-CoV-2 Virus. Appl. Sci..

[55] Behl T., Kaur I., Aleya L., Sehgal A., Singh S., Sharma N., Bhatia S., Al-Harrasi A., Bungau S. (2022). CD147-Spike Protein Interaction in COVID-19: Get the Ball Rolling with a Novel Receptor and Therapeutic Target. Sci. Total Environ..

[56] Boschi C., Scheim D.E., Bancod A., Millitello M., Le Bideau M., Colson P., Fantini J., Scola B. (2022). La SARS-CoV-2 Spike Protein Induces Hemagglutination: Implications for COVID-19 Morbidities and Therapeutics and for Vaccine Adverse Effects. Int. J. Mol. Sci..

[57] Bogomol’tsev B.P., Deviatkin A.V. (2003). Clinical Implications of Impaired Microcirculation and Hemodynamics in Acute Respiratory Viral Infections and Their Pharmacological Correction. Klin. Med..

[58] Vicaut E., Hou X., Decuypère L., Taccoen A., Duvelleroy M. (1994). Red Blood Cell Aggregation and Microcirculation in Rat Cremaster Muscle. Int. J. Microcirc. Clin. Exp..

[59] Ehrly A.M. (1986). Erythrocyte Aggregation in Clinical Medicine. Klin. Wochenschr..

[60] Durussel J.J., Berthault M.F., Guiffant G., Dufaux J. (1998). Effects of Red Blood Cell Hyperaggregation on the Rat Microcirculation Blood Flow. Acta Physiol. Scand..

[61] McHedlishvili G., Varazashvili M., Gobejishvili L. (2002). Local RBC Aggregation Disturbing Blood Fluidity and Causing Stasis in Microvessels. Clin. Hemorheol. Microcirc..

[62] Ballering A.V., van Zon S.K.R., olde Hartman T.C., Rosmalen J.G.M. (2022). Persistence of Somatic Symptoms after COVID-19 in the Netherlands: An Observational Cohort Study. Lancet.

[63] Abdelnour L., Eltahir Abdalla M., Babiker S. (2020). COVID 19 Infection Presenting as Motor Peripheral Neuropathy. J. Formos. Med. Assoc..

[64] Lowe G.D.O., Lee A.J., Rumley A., Price J.F., Fowkes F.G.R. (1997). Blood Viscosity and Risk of Cardiovascular Events: The Edinburgh Artery Study. Br. J. Haematol..

[65] Viallat A., Abkarian M. (2014). Red Blood Cell: From Its Mechanics to Its Motion in Shear Flow. Int. J. Lab. Hematol..

[66] Zhbanov A., Yang S. (2015). Effects of Aggregation on Blood Sedimentation and Conductivity. PLoS ONE.

[67] Yalcin O., Uyuklu M., Armstrong J.K., Meiselman H.J., Baskurt O.K. (2004). Graded Alterations of RBC Aggregation Influence in Vivo Blood Flow Resistance. Am. J. Physiol.-Heart Circ. Physiol..

[68] Murali C., Nithiarasu P. (2017). Red Blood Cell (RBC) Aggregation and Its Influence on Non-Newtonian Nature of Blood in Microvasculature. J. Model. Mech. Mater..

[69] Barshtein G., Ben-Ami R., Yedgar S. (2007). Role of Red Blood Cell Flow Behavior in Hemodynamics and Hemostasis. Expert Rev. Cardiovasc. Ther..

[70] Pries A.R., Secomb T.W., Gaehtgens P. (1996). Biophysical Aspects of Blood Flow in the Microvasculature. Cardiovasc. Res..

[71] Yalcin O., Ulker P., Yavuzer U., Meiselman H.J., Baskurti O.K. (2008). Nitric Oxide Generation by Endothelial Cells Exposed to Shear Stress in Glass Tubes Perfused with Red Blood Cell Suspensions: Role of Aggregation. Am. J. Physiol.-Heart Circ. Physiol..

[72] Druzak S., Iffrig E., Roberts B.R., Zhang T., Fibben K.S., Sakurai Y., Verkerke H.P., Rostad C.A., Chahroudi A., Schneider F. (2023). Multiplatform Analyses Reveal Distinct Drivers of Systemic Pathogenesis in Adult versus Pediatric Severe Acute COVID-19. Nat. Commun..

[73] Mehta J.L., Calcaterra G., Bassareo P.P. (2020). COVID-19, Thromboembolic Risk, and Virchow’s Triad: Lesson from the Past. Clin. Cardiol..

[74] Ami R.B., Barshtein G., Zeltser D., Goldberg Y., Shapira I., Roth A., Keren G., Miller H., Prochorov V., Eldor A. (2001). Parameters of Red Blood Cell Aggregation as Correlates of the Inflammatory State. Am. J. Physiol.-Heart Circ. Physiol..

[75] Pancani R., Villari L., Foci V., Parri G., Barsotti F., Patrucco F., Malerba M., Vincenti R., Carrozzi L., Celi A. (2021). Lower Limb Deep Vein Thrombosis in COVID-19 Patients Admitted to Intermediate Care Respiratory Units. Thromb. Res..

[76] Mumoli N., Dentali F., Conte G., Colombo A., Capra R., Porta C., Rotiroti G., Zuretti F., Cei M., Tangianu F. (2022). Upper Extremity Deep Vein Thrombosis in COVID-19: Incidence and Correlated Risk Factors in a Cohort of Non-ICU Patients. PLoS ONE.

[77] Gul M.H., Htun Z.M., de Jesus Perez V., Suleman M., Arshad S., Imran M., Vyasabattu M., Wood J.P., Anstead M., Morris P.E. (2023). Predictors and Outcomes of Acute Pulmonary Embolism in COVID-19; Insights from US National COVID Cohort Collaborative. Respir. Res..

[78] Yu F.T.H., Armstrong J.K., Tripette J., Meiselman H.J., Cloutier G. (2011). A Local Increase in Red Blood Cell Aggregation Can Trigger Deep Vein Thrombosis: Evidence Based on Quantitative Cellular Ultrasound Imaging. J. Thromb. Haemost..

[79] Ionescu D.A., Ghiţescu M.I., Andronescu S., Marcu I. (1983). Contribution of the Erythrocytes Physical Qualities (Deformability and Aggregability) to the Viscoelastic Properties of the Blood Clot in Patients with Acute Cerebral Thrombosis. Neurol. Psychiatr..

[80] Wang Q., Zennadi R. (2020). Oxidative Stress and Thrombosis during Aging: The Roles of Oxidative Stress in RBCs in Venous Thrombosis. Int. J. Mol. Sci..

[81] Weisel J.W., Litvinov R.I. (2019). Red Blood Cells: The Forgotten Player in Hemostasis and Thrombosis. J. Thromb. Haemost..

[82] Nash G.B., Watts T., Thornton C., Barigou M. (2008). Red Cell Aggregation as a Factor Influencing Margination and Adhesion of Leukocytes and Platelets. Clin. Hemorheol. Microcirc..

[83] Goldsmith H.L., Bell D.N., Spain S., McIntosh F.A. (1999). Effect of Red Blood Cells and Their Aggregates on Platelets and White Cells in Flowing Blood. Biorheology.

[84] Sun J., Liu H., Zhang H. (2006). Influence of Erythrocyte Aggregation on Leukocyte Margination in Postcapillary Expansions: A Lattice Boltzmann Analysis. Phys. A Stat. Mech. Appl..

[85] Stroobach M., Haya L., Fenech M. (2019). Effects of Red Blood Cell Aggregation on Microparticle Wall Adhesion in Circular Microchannels. Med. Eng. Phys..

[86] Sprague B., Chesler N.C., Magness R.R. (2010). Shear Stress Regulation of Nitric Oxide Production in Uterine and Placental Artery Endothelial Cells: Experimental Studies and Hemodynamic Models of Shear Stress Forces on Endothelial Cells. Int. J. Dev. Biol..

[87] Kabirian F., Amoabediny G., Haghighipour N., Salehi-Nik N., Zandieh-Doulabi B. (2015). Nitric Oxide Secretion by Endothelial Cells in Response to Fluid Shear Stress, Aspirin, and Temperature. J. Biomed. Mater. Res. A.

[88] Sriram K., Laughlin J.G., Rangamani P., Tartakovsky D.M. (2016). Shear-Induced Nitric Oxide Production by Endothelial Cells. Biophys. J..

[89] Baskurt O.K., Yalcin O., Ozdem S., Armstrong J.K., Meiselman H.J. (2003). Modulation of Endothelial Nitric Oxide Synthase Expression by Red Blood Cell Aggregation. Am. J. Physiol.-Heart Circ. Physiol..

[90] Freedman J.E., Loscalzo J. (2003). Nitric Oxide and Its Relationship to Thrombotic Disorders. J. Thromb. Haemost..

[91] Cicha I., Suzuki Y., Tateishi N., Maeda N. (2003). Changes of RBC Aggregation in Oxygenation-Deoxygenation: PH Dependency and Cell Morphology. Am. J. Physiol.-Heart Circ. Physiol..

[92] Ulker P., Meiselman H.J., Baskurt O.K. (2010). Nitric Oxide Generation in Red Blood Cells Induced by Mechanical Stress. Clin. Hemorheol. Microcirc..

[93] Rovas A., Osiaevi I., Buscher K., Sackarnd J., Tepasse P.R., Fobker M., Kühn J., Braune S., Göbel U., Thölking G. (2021). Microvascular Dysfunction in COVID-19: The MYSTIC Study. Angiogenesis.

[94] Yamaoka-Tojo M. (2020). Vascular Endothelial Glycocalyx Damage in COVID-19. Int. J. Mol. Sci..

[95] Zha D., Fu M., Qian Y. (2022). Vascular Endothelial Glycocalyx Damage and Potential Targeted Therapy in COVID-19. Cells.

[96] Becker B.F., Jacob M., Leipert S., Salmon A.H.J., Chappell D. (2015). Degradation of the Endothelial Glycocalyx in Clinical Settings: Searching for the Sheddases. Br. J. Clin. Pharmacol..

[97] Tarbell J.M., Pahakis M.Y. (2006). Mechanotransduction and the Glycocalyx. J. Intern. Med..

[98] Bosek M., Ziomkowska B., Pyskir J., Wybranowski T., Pyskir M., Cyrankiewicz M., Napiórkowska M., Durmowicz M., Kruszewski S. (2022). Relationship between Red Blood Cell Aggregation and Dextran Molecular Mass. Sci. Rep..

[99] Zuin M., Barco S., Giannakoulas G., Engelen M.M., Hobohm L., Valerio L., Vandenbriele C., Verhamme P., Vanassche T., Konstantinides S.V. (2023). Risk of Venous Thromboembolic Events after COVID-19 Infection: A Systematic Review and Meta-Analysis. J. Thromb. Thrombolysis.

[100] Giannis D., Allen S.L., Tsang J., Flint S., Pinhasov T., Williams S., Tan G., Thakur R., Leung C., Snyder M. (2021). Postdischarge Thromboembolic Outcomes and Mortality of Hospitalized Patients with COVID-19: The CORE-19 Registry. Blood J. Am. Soc. Hematol..

[101] World Health Organization (2020). Clinical Management of Severe Acute Respiratory Infection (SARI) When COVID-19 Disease Is Suspected: Interim Guidance.

[102] Bosek M., Szołna-Chodór A., Antonova N., Grzegorzewski B. (2018). The Fractal Dimension of Red Blood Cell Aggregates in Dextran 70 Solutions. Opt. Appl..

[103] Richardson J.F., Zaki W.N. (1997). Sedimentation and Fluidisation: Part I. Chem. Eng. Res. Des..

[104] Johnson C.P., Li X., Logan B.E. (1996). Settling Velocities of Fractal Aggregates. Environ. Sci. Technol..

